# First‐trimester exposure to tofacitinib in ulcerative colitis: A case report of a healthy newborn and literature review

**DOI:** 10.1002/ccr3.8764

**Published:** 2024-04-12

**Authors:** Arteen Arzivian, Eva Zhang, Robyn Laube, Rupert Leong

**Affiliations:** ^1^ Department of Gastroenterology and Hepatology Macquarie University Hospital Sydney New South Wales Australia; ^2^ Faculty of Medicine, Health and Human Sciences, Macquarie Medical School Macquarie University Sydney New South Wales Australia

**Keywords:** case report, inflammatory bowel diseases, Janus kinase inhibitors, pregnancy, tofacitinib, ulcerative colitis

## Abstract

Tofacitinib is contraindicated in pregnancy. We present a patient with ulcerative colitis on tofacitinib who had an unplanned pregnancy. Tofacitinib was ceased, switched to vedolizumab, and she gave birth to a healthy newborn at term. Case reports of reassuring outcomes provide real‐world data that assists decision‐making for future patients.

## Graphical abstract

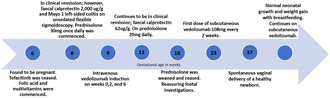

## 1 INTRODUCTION

Tofacitinib is approved for moderately to severely active ulcerative colitis (UC).^1^ Animal studies showed increased pregnancy loss, lower foetal weight, and congenital malformations; the doses used were much higher than the maximum recommended dose of 10 mg twice daily in humans.^2^ The only evidence for use in pregnancy in patients with inflammatory bowel disease (IBD) is from the registration studies, which revealed 11 maternal exposures with foeto‐maternal adverse events rates comparable to the general population and patients with similar conditions not taking tofacitinib; however, the number of cases was small, the comparison was indirect, and subjects received pre‐emptive pregnancy testing at every study visit to permit early treatment cessation.^3^ Until further advice is available, tofacitinib should be avoided in pregnancy.^4^

We present a case of exposure to tofacitinib in the first trimester until 6 weeks of gestation in a patient with UC.

## 2 CASE HISTORY/EXAMINATION

A woman in her 20s with left‐sided UC (Montreal E2) treated with tofacitinib 10 mg twice daily presented to our clinic after having a positive pregnancy test. Her beta‐human chorionic gonadotropin level was 18,477 IU/L; an ultrasound confirmed pregnancy with a gestational age of 6 weeks.

She was diagnosed with UC at the age of 8 years and was initially treated with mesalazine until adolescence. Azathioprine was added due to active colitis following the transition to the adult IBD clinic but was ceased after the development of acute pancreatitis. She developed severe allergic reactions to both infliximab and adalimumab. She responded well to intravenous vedolizumab 300 mg 8‐weekly. However, the patient stopped attending vedolizumab infusions due to poor venous access in 2018 before the availability of subcutaneous vedolizumab. Six months after non‐adherence, she presented with acute severe UC that failed to respond to intravenous hydrocortisone 100 mg four times daily. She responded to intravenous ciclosporin 4 mg/kg infusions but was complicated by a seizure necessitating ciclosporin cessation. Tofacitinib 10 mg twice daily was initiated, which led to significant clinical improvement. When tofacitinib dose was reduced to 5 mg twice daily, she relapsed with an increase of fecal calprotectin to 907 μg/g. Flexible sigmoidoscopy showed Mayo 1 left‐sided colitis. Tofacitinib dose was increased to 10 mg twice daily, which recaptured remission. The patient was in a stable relationship and was advised against pregnancy while taking tofacitinib, and she agreed to use contraception.

## 3 DIFFERENTIAL DIAGNOSIS, INVESTIGATIONS, AND TREATMENT

Six months into maintenance treatment on tofacitinib, the patient performed a pregnancy test due to amenorrhea. Despite being adherent to the combined oral contraceptive pills, the pregnancy test returned a positive result. First‐trimester ultrasound confirmed the gestational age of 6 weeks. Tofacitinib was ceased immediately. She and her partner were consulted regarding the potential foeticidal and teratogenic effects of tofacitinib and the lack of human studies on pregnancy outcomes. They decided to continue the pregnancy. She was commenced on folic acid 5 mg daily and oral pre‐conception multi‐vitamin supplement tablets and referred to a high‐risk pregnancy clinic. After cessation of tofacitinib, despite being in clinical remission, a follow‐up fecal calprotectin had increased to 2000 μg/g. An unsedated flexible sigmoidoscopy showed Mayo 1 left‐sided colitis at 8 weeks gestation. Prednisolone 30 mg daily was commenced. Due to her previous remission on vedolizumab and the recent introduction of subcutaneous maintenance vedolizumab, she agreed to intravenous induction of 300 mg at weeks 0, 2, and 6, followed by subcutaneous vedolizumab 108 mg every other week. At 11 weeks of gestation, she remained in clinical remission on prednisolone 20 mg daily, and her fecal calprotectin decreased to 62 μg/g. First‐trimester noninvasive prenatal tests for foetal aneuploidy and nuchal translucency examination were unremarkable. Prednisolone was successfully tapered over 2 months, and she continued subcutaneous vedolizumab throughout her pregnancy. During the pregnancy period, she reviewed regularly every 8–12 weeks in the IBD and high‐risk pregnancy clinics.

## 4 OUTCOME AND FOLLOW‐UP

She entered labour spontaneously at 37 weeks and delivered vaginally to a healthy boy with an Apgar score of 10, weighing 2.265 kg (maternal pre‐pregnancy weight was 48 kg). Neonatal jaundice was treated with phototherapy and resolved. Postpartum advice was encouragement to breastfeed, continue subcutaneous vedolizumab, and avoid rotavirus vaccination due to in‐utero exposure to vedolizumab. Six months postpartum follow‐up, she continued breastfeeding her newborn child. She was in clinical and biochemical remission on subcutaneous vedolizumab therapy; she is booked for a repeat colonoscopy for an endoscopic assessment of the disease activity. The newborn's growth and weight gain were normal.

**FIGURE 1:**
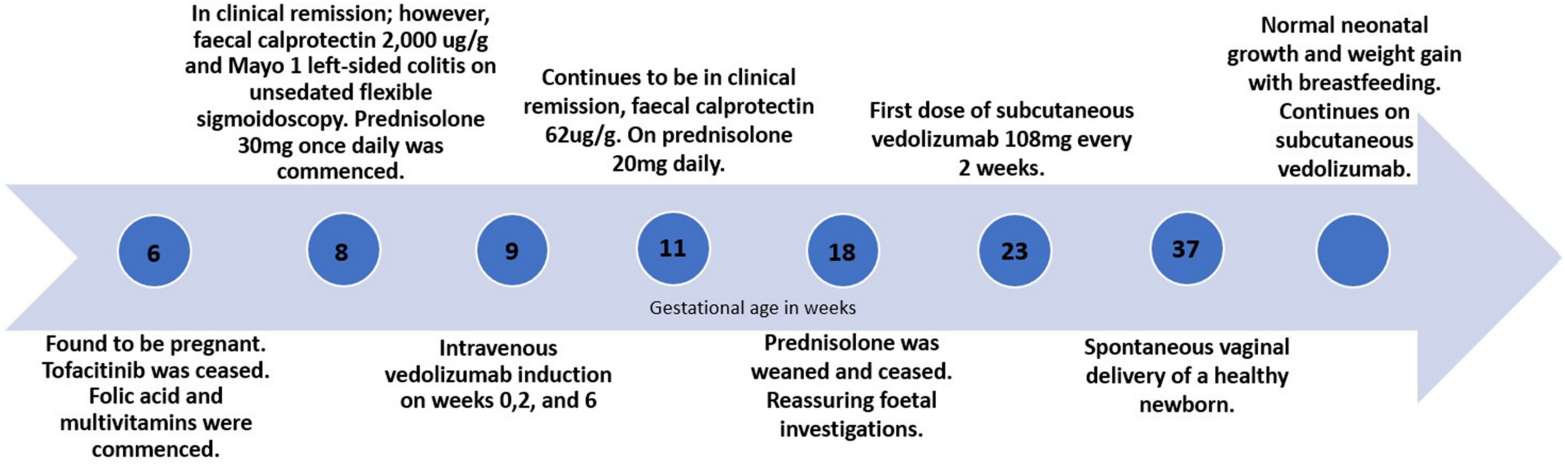
The timeline of events during pregnancy in our patient with ulcerative colitis who was in remission on tofacitinib 10 mg twice daily and found to be pregnant at 6 weeks of gestation.

Figure 1 demonstrates the timeline of events during our patient's pregnancy.

## 5 DISCUSSION

Tofacitinib is an oral, small‐molecule Janus kinase (JAK) inhibitor. It is a second‐generation JAK inhibitor that selectively inhibits JAK1 and JAK3.^5^ It was first approved for moderately to severely active rheumatoid arthritis in 2012, followed by approval for psoriatic arthritis, active polyarticular course juvenile idiopathic arthritis and active ankylosing spondylitis 2021.^6^, ^7^, ^8^, ^9^ Tofacitinib was approved in 2018 for induction and maintenance of moderately to severely active UC.^1^

Despite being approved and used for rheumatological conditions for more than a decade, there is a paucity of real‐life data regarding its use in pregnancy. A review of the pregnancy cases identified in the randomized clinical trials of tofacitinib for rheumatoid arthritis and psoriatic arthritis showed a total number of 47 women who became pregnant during the trials, 33 on tofacitinib monotherapy and 13 on tofacitinib and methotrexate combination and one patient had a blinded therapy. There were 25 healthy newborns 53.2%, eight medical terminations 17% (four monotherapy, three combination therapy and one blinded therapy), seven spontaneous abortions 14.9% (four monotherapy and three combination therapy), one congenital pulmonary valve stenosis 2.1% (on monotherapy), no foetal deaths reported, and six pending or lost to follow‐up 12.8%. The rate of birth defects and spontaneous abortions was comparable to the general population and to patients with similar conditions who are not using tofacitinib.^10^ Due to the small sample size, retrospective study, and the lack of a randomized clinical trial, tofacitinib is still not considered safe to treat rheumatological conditions in pregnancy.^11^, ^12^

Tofacitinib use for IBD in pregnancy is not recommended, and cessation for 4–8 weeks is advised before conception; this is based on its foeticidal and teratogenic potential in animal studies and due to the lack of evidence of use in humans.^4^, ^13^ It is assumed that tofacitinib can cross the placenta due to its small molecular size, although this has yet to be formally tested and studied. Animal studies showed increased loss of pregnancy and lower mean foetal body weight. Congenital malformations included membranous ventricular septal defects, anasarca, skeletal, soft tissue, and cranial deformities. The doses used in these studies were 73 times (in rats) and 6.3 times (in rabbits) the maximum recommended dose of 10 mg twice daily in humans.^2^

UC affects females in the reproductive age group with fewer biological therapies available, which increases the need for newer advanced therapies like tofacitinib, which is used in higher doses of 10 mg twice daily in UC compared to rheumatoid arthritis. Continuation of medical treatment for UC during pregnancy is recommended due to the increased risk of active disease and poor pregnancy outcomes associated with interruption of treatment.^4^ In the interventional studies of tofacitinib in UC, there were 11 cases of maternal exposure, all patients were exposed during the first trimester, specific gestational age at the time of exposure could not be determined, and all patients except one were on 10 mg twice a day dose. Four healthy newborns 36.4%, two spontaneous abortions 18.2%, and two medication terminations 18.2% were reported, and three were pending or lost to follow‐up 27.2%.^14^ Tofacitinib level in breast milk was found to exceed maternal serum levels.^15^ Tofacitinib should be avoided in breastfeeding.^4^

In 19 healthy female volunteers, tofacitinib effect on the efficacy and pharmacokinetics of oral contraceptives was evaluated, and there was no evidence of a drug–drug interaction to suggest decreased efficacy of oral contraceptive tablets or the need for dose adjustment.^16^

There is a lack of case reports of tofacitinib exposure in pregnancy. A case of a 40‐year‐old patient with psoriatic arthritis who was exposed to tofacitinib was reported; the patient delivered a healthy newborn.^17^ However, she was treated on a lower dose of tofacitinib of 5 mg twice daily and had a shorter exposure to tofacitinib while pregnant than our patient.

Real‐life case reports of exposure and outcome of tofacitinib in pregnancy add to the limited available real‐life evidence. Post‐marketing registries are essential to obtain larger experience that might modify clinical practice guidelines in the future.^18^

## AUTHOR CONTRIBUTIONS

**Arteen Arzivian:** Conceptualization; data curation; resources; software; writing – original draft. **Eva Zhang:** Project administration; validation; writing – review and editing. **Robyn Laube:** Data curation; project administration; resources; validation; writing – review and editing. **Rupert Leong:** Conceptualization; project administration; resources; supervision; writing – review and editing.

## FUNDING INFORMATION

This case report did not receive financial support.

## CONFLICT OF INTEREST STATEMENT

Rupert W. Leong reports advisory board membership of AbbVie, Aspen, BMS, Celgene, Celltrion, Chiesi, Ferring, Glutagen, Hospira, Janssen, Lilly, MSW, Novartis, Pfizer, Prometheus Biosciences, Takeda and research grant support from McCusker Charitable Foundation, Joanna Tiddy grant University of Sydney, Celltrion, Shire, Janssen, Takeda, Gastroenterological Society of Australia, MRFF, NHMRC, Gutsy Group, Pfizer. All the other authors have no conflict of interest to declare.

## ETHICS STATEMENT

This case report was approved by the Macquarie Health Clinical Innovation and Audit Committee. Reference number: MQCIAC2023009.

## CONSENT

The patient has provided written consent to publish this case report and all accompanying details of her health issue.

## Data Availability

The data in this case report are not publicly available due to containing information that could compromise the patient's privacy but are available from the corresponding author AA upon reasonable request.
